# The chloroplast genomes of two medicinal species (*Veronica anagallis-aquatica* L*.* and *Veronica undulata* Wall.) and its comparative analysis with related *Veronica* species

**DOI:** 10.1038/s41598-024-64896-7

**Published:** 2024-06-17

**Authors:** Yonglin Hai, Yan Qian, Meihua Yang, Yue Zhang, Huimei Xu, Yongcheng Yang, Conglong Xia

**Affiliations:** 1 College of Pharmacy, Dali University, Dali, 671000 China; 2 Key Laboratory of Yunnan Provincial Higher Education Institutions for Development of Yunnan Daodi Medicinal Materials Resources, Dali, 671000 China

**Keywords:** Genetics, Molecular biology

## Abstract

*Veronica anagallis-aquatica* L. and *Veronica undulata* Wall. are widely used ethnomedicinal plants in China. The two species have different clinical efficacies, while their extremely similar morphology and unclear interspecific relationship make it difficult to accurately identify them, leading to increased instances of mixed usage. This article reports on the complete chloroplast genomes sequence of these two species and their related *Veronica* species to conduct a comparative genomics analysis and phylogenetic construction. The results showed that the chloroplast (cp) genomes of *Veronica* exhibited typical circular quadripartite structures, with total lengths of 149,386 to 152,319 base pairs (bp), and GC content of 37.9 to 38.1%, and the number of genes was between 129–134. The total number of simple sequence repeats (SSRs) in *V. anagallis-aquatica* and *V. undulata* is 37 and 36, while *V. arvensis* had the highest total number of 56, predominantly characterized by A/T single bases. The vast majority of long repeat sequence types are forward repeats and palindromic repeats. Selective Ka/Ks values showed that three genes were under positive selection. Sequence differences often occur in the non-coding regions of the large single-copy region (LSC) and small single-copy region (SSC), with the lowest sequence variation in the inverted repeat regions (IR). Seven highly variable regions (*trnT-GGU-psbD*, *rps8-rpl16*, *trnQ-UUG*, *trnN-GUU-ndhF*, *petL*, *ycf3*, and *ycf1*) were detected, which may be potential molecular markers for identifying *V. anagallis-aquatica* and *V. undulata.* The phylogenetic tree indicates that there is a close genetic relationship between the genera *Veronica* and *Neopicrorhiza,* and *V. anagallis-aquatica* and *V. undulata* are sister groups. The molecular clock analysis results indicate that the divergence time of *Veronica* may occur at ∼ 9.09 Ma, and the divergence time of these two species occurs at ∼ 0.48 Ma. It is speculated that climate change may be the cause of *Veronica* species diversity and promote the radiation of the genus. The chloroplast genome data of nine *Veronica* specie provides important insights into the characteristics and evolution of the chloroplast genome of this genus, as well as the phylogenetic relationships of the genus *Veronica*.

## Introduction

**Figure 1:**
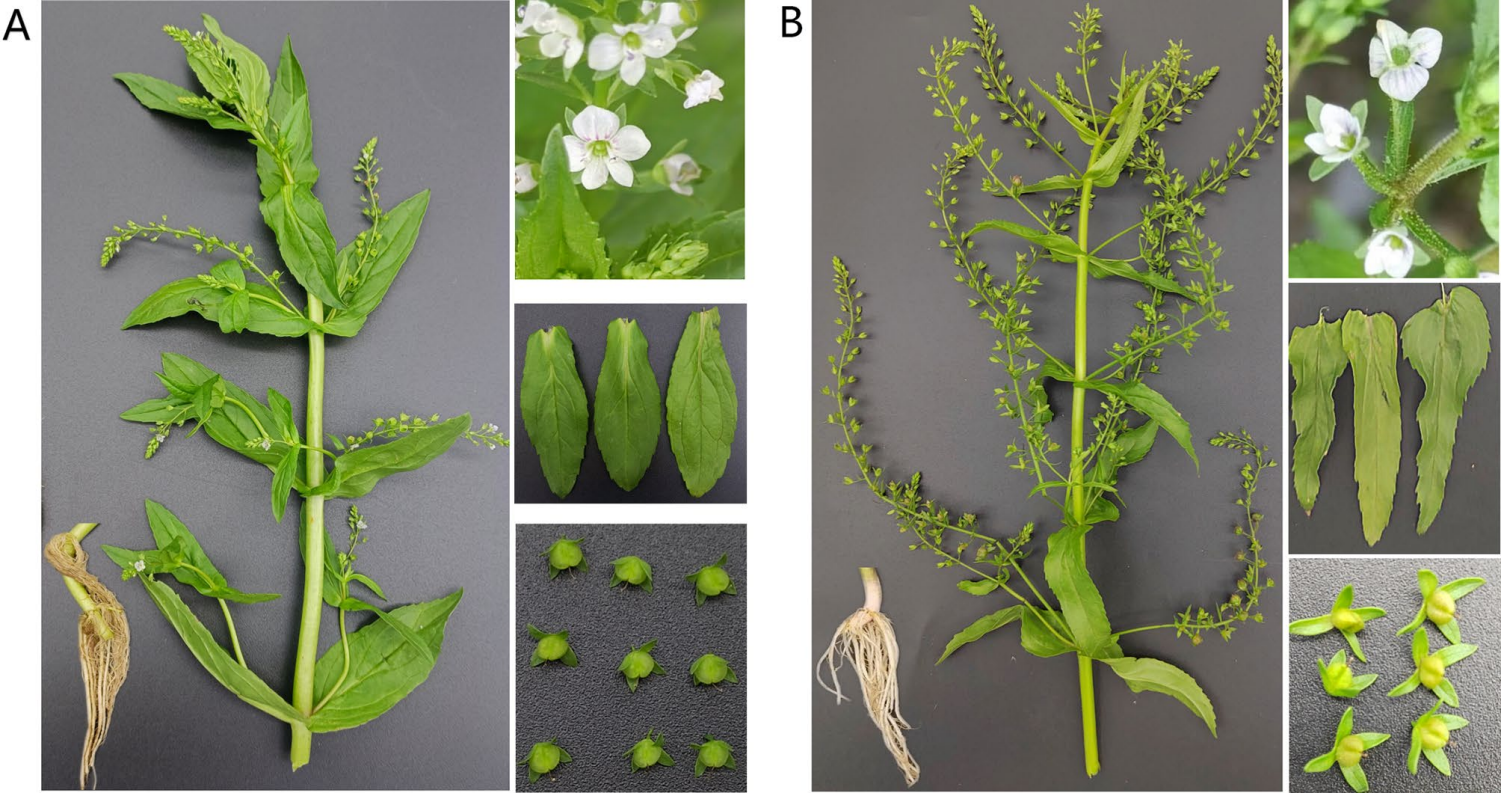
Plant morphology of *V. anagallis-aquatica* (**A**) and *V. undulata* (**B**). (The pictures including roots, stems, leaves, flowers, and fruits.).

*Veronica anagallis-aquatica* L. is a kind of *Veronica* plant belonging to the family Plantaginaceae, with a long history of ethnomedicinal use, widely distributed in China throughout its provinces north and southwest of the Yangtze River, and mainly in temperate regions of Asia and Europe^1^. Its fruit is abnormally swollen due to insect parasitism, and the whole plant is harvested, washed, and used fresh or dried before the parasitic insects escape. This plant with insect galls is used clinically in traditional Mongolian medicine (TMM) to treat edema and arthralgia due to its diuretic and edema-reducing effects^2^. *Veronica undulata* Wall. is widely distributed throughout China. It has blood-activating and pain-relieving effects as mentioned in the *Processing Standards of Traditional Chinese Medicine (TCM) Decoction Pieces in Shanghai* (2018 Edition). Clinically, it is employed in the treatment of conditions such as hemoptysis, stomach pain, rheumatic pain, dysmenorrhea, carbuncles, and swelling^3^. Previous studies have shown that *V. anagallis-aquatica* and *V. undulata* have different efficacies and applications, but have very similar morphological characteristics^4^. *V. anagallis-aquatica* exhibits a slightly greater height compared to *V. undulata*, with leaves mostly elliptical or oval shaped, and almost no glandular hairs on the whole. In contrast, the leaves of *V. undulata* are mostly strip lanceolate, the leaf margin has sharp serrate, and the stem and inflorescence axis have needle glandular hairs^5^. Additionally, traditional morphological identification methods cannot effectively identify them. Ancient materia medica provide limited distinctions between these two species, which cause confusion since they are closely related species in the same family and genus^4^ (Fig. 1).

Approximately 250 species of plants in the genus *Veronica* exist worldwide, mainly distributed throughout Eurasia. Moreover, 61 of these species are spread throughout China, mainly in its southwestern mountains, while up to 27 species are currently recorded as having medicinal properties^6^. Extensive research on the chemical constituents of this genus has resulted in phytochemists isolating and identifying over 260 compounds from its plants, including iridoid glycosides, p-Phenylethanoside, and flavonoids^7^. Research on modern contemporary activity indicates that this genus exhibits anti-inflammatory, antibacterial, antioxidant, anti-tumor, in vitro coagulation, antitussive, and liver-protective effects^8–10^ The traditional identification method of *Veronica* plants is mainly based on the morphological characteristics of their leaf shape, petiole, flower stalk, fruit stalk length, flower color and size, fruit shape, petiole root structure, as well as the number of stomata on the upper and lower epidermis of its leaves^11,12^. However, each distribution area has a different environment since some species are widely distributed. To adapt to local ecological environments, the related traits of these species are discontinuous between each group^12^. In addition, due to the short time of species differentiation and gene exchange between closely related species, some species have a vague interspecific relationship^13^. Therefore, it is difficult to accurately identify the species of this genus only by relying on traditional morphological methods.

In addition, the Angiosperm phylogeny classification of flowering plants (APG IV)^14^ system removed *Veronica* from Scrophulariaceae and moved it to Plantaginaceae. Many scholars have conducted extensive studies and analyses of it, taking into account various aspects such as biochemistry, molecular biology, and pharmacognosy. However, the determination of the phylogenetic position of the genus *Veronica* remains very controversial, and scholars have expressed diverse views on this issue. For example, Soren Rosendal Jensen et al. detected non-flavonoid glycosides in *Veronica* and its related genera, and combined ITS, plastid *trnL-F*, *rps16* gene region, and DNA intron region to investigate the phylogenetic relationship of the genus *Veronica* and its related genera. The phylogenetic relationship between *Veronica* and *Paederota* is the closest, followed by *Picrorhiza*^15^. Song Xiaolin conducted a study on the species related to *Veronica* and its associated genera by analyzing their morphological characteristics. It was found that *Veronica* exhibited greater resemblance to *Scrophularia* by leaf epidermal micromorphology, and *Veronica* was more similar to *Plantago* according to seed shape and seed coat micromorphology. Finally, cluster analysis of 28 traits of 18 species of *Veronica* and its related plants showed that *Veronica* was closely related to *Scrophularia*^16^. The phylogenetic relationship of *Rehmannia* and its related groups was studied by chloroplast genome sequences. It was found that *Veronica* and *Rehmannia*, *Digitalis,* and *Plantago* constitute a monophyletic system, which supports the treatment of related families and genera by APG system^17,18^. *Veronica* plants have significant potential ecological^19,20^ environmental^21^ and medicinal^7,22–24^ value. Therefore, their taxonomic issues must be further explored.

With the development of sequencing technology, molecular biology and other disciplines, using appropriate molecular markers to analyze closely related interspecies and intraspecific sequences is an effective method to solve the problems of interspecies relationships and classification in systems^25–27^. Chloroplasts (cp) are an essential organelle in plant cells intended for photosynthesis and other functions, including the synthesis of starch, fatty acids, pigments, and amino acids^28^. Recently, the cp genome has provided more information about genetic variations and significantly improved the phylogenetic resolution of species. It has been widely applied when developing molecular markers to classify medicinal plants and implemented in phylogenetic studies^29,30^. In most angiosperms, this genome is usually double-stranded and circular^31^. It consists of a small single copy region (SSC), a large single copy region (LSC), and a pair of inverted repeat regions (IR)^32^. The cp genome has a highly conserved structure, gene content, and typically low level of DNA sequence variation compared to nuclear and mitochondrial genomes, and many studies have reported that it is expected to serve as a super barcode to identify closely related species^33,34^.

Our study aims to: (i) contribute a fully-sequenced cp genome of *Veronica* species, of which *V. anagallis-aquatica* was reported for the first time, and expand our understanding of the overall structure of these genomes. (ii) *V. anagallis-aquatica* and *V. undulata* have very similar morphologies. By comparing and analyzing the structural characteristics of the whole cp genome of these two species, potential molecular markers were screened to distinguish them. (iii) To explain the phylogenetic evolution of the two species and their genus, and to provide a reference for the traditional taxonomy as well as further application and conservation of the medicinal plants in *Veronica*.

## Results

### The structure and characteristics of the cp genome of *Veronica*

**Figure 2:**
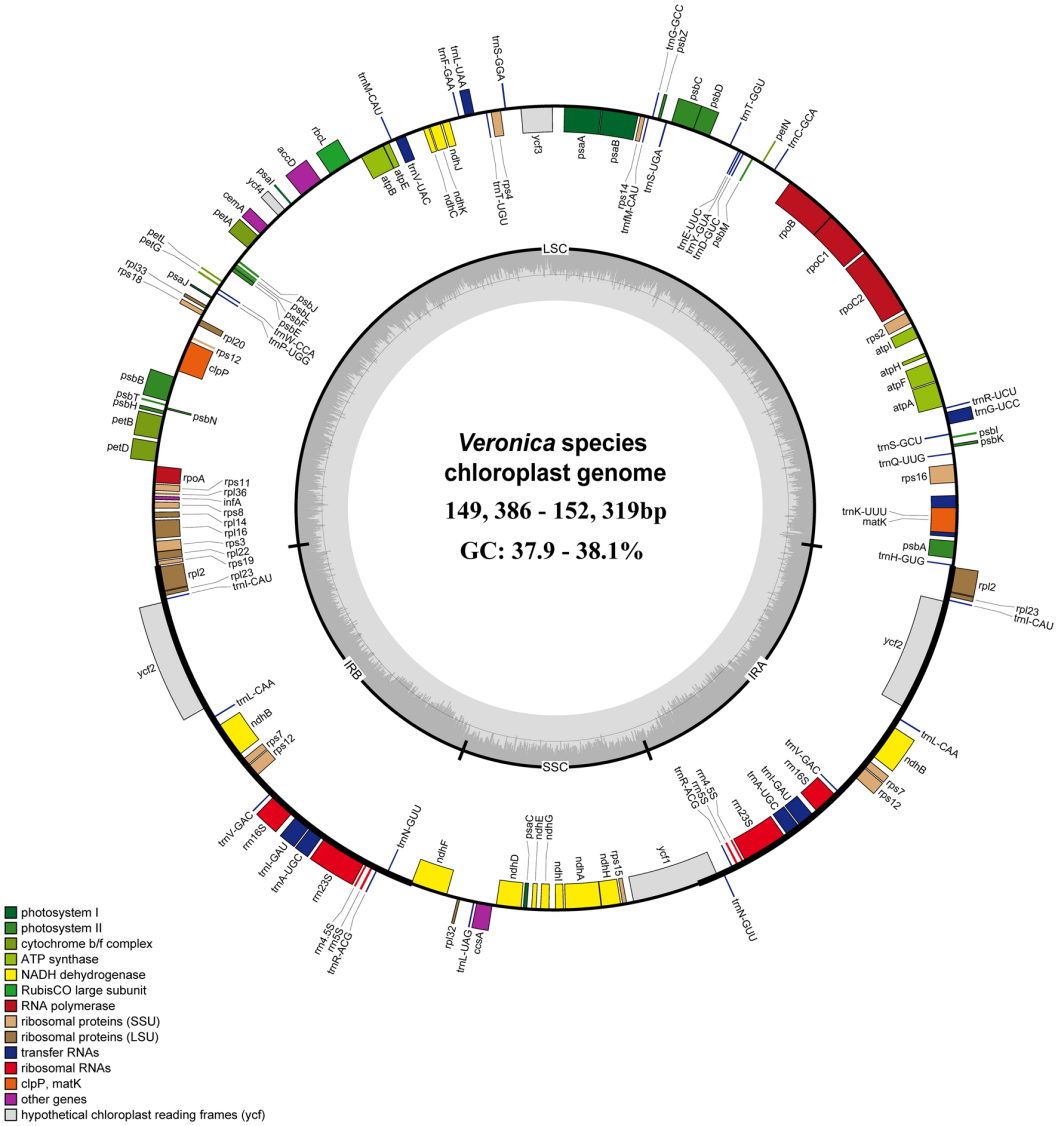
Physical map of cp genome of 9 *Veronica* species. Genes inside the circle are transcribed in the clockwise direction, while genes outside the circle are transcribed in the counterclockwise direction. The dark grey area in the inner circle reflects the GC content of the cp genome, whereas the light grey area represents the AT content. The genes that belong to distinct functional groupings are shown by different color blocks. The quadripartite structure is also reported as: *LSC* large single copy, *SSC* small single copy, *IRA* inverted repeat A, *IRB* inverted repeat B.

*V. undulata* (OQ564497.1) was selected as the reference gene of the *V. anagallis-aquatica* and *V. undulata* for annotation. The complete cp genomes of these species were annotated to obtain 130 genes, including 85 protein-coding genes, 37 tRNA genes, and 8 rRNA genes, which were mainly categorized as photosynthesis genes, replication genes, unknown functional genes, and acetyl-CoA carboxylase subunits (*accD*), mature enzyme genes (*matK*), transcription initiation factors (*infA*), and other genes. In addition, 17 genes were found to contain introns in the cp genomes of both species (Table S1), of which nine protein-coding genes and six tRNA-coding genes contained one intron, while the *ycf3* and *clpP* protein-coding genes contained two introns. The length difference between *Veronica* is significant, ranging from 149,386 to 152,319 base pairs (bp), and the cpDNA showed a typical tetrad ring structure (Fig. 2). These both consisted of a pair of inverted repeats (IR) and a large single copy region as well as a small single-copy. The difference in total GC content within the 9 species of the *Veronica* genus is relatively small, ranging from 37.9% to 38.1%. Additionally, the number of genes was 129–134. Significantly, the GC content in the IR region of 9 species is higher than that in the LSC and SSC regions, with the SSC region having the lowest content (Table 1).

**Table 1:** Basic information of cp genome of *Veronica* species.

	*V. anagallis-aquatica*	*V. undulata*	*V. agrestis*	*V. arvensis*	*V. chamaedrys*	*V. eriogyne*	*V. nakaiana*	*V. ovata* subsp.* kiusiana*	*V. polita*
Total length (bp)	151,277	151,201	150,197	149,386	150,562	151,083	152,319	152,249	150,191
LSC length (bp)	82,598	82,667	81,850	82,004	82,008	82,302	83,195	83,187	81,847
SSC length (bp)	17,539	17,402	17,417	17,482	17,490	17,449	17,704	17,704	17,414
IR length (bp)	51,140	51,132	50,930	49,900	51,064	51,332	51,385	51,358	50,930
Total number of genes	130	130	130	133	129	134	133	133	134
protein-coding genes	85	85	85	88	84	88	88	88	86
tRNA gene	37	37	37	37	37	38	37	37	38
rRNA gene	8	8	8	8	8	8	8	8	8
GC content (%)	38.1	38.1	37.9	37.9	38.0	38.0	37.9	38.0	37.9
GC content of LSC (%)	36.2	36.2	36.0	36.0	36.2	36.2	36.0	36.1	36.0
GC content of IR (%)	43.2	43.2	43.2	43.0	43.1	43.1	42.2	43.2	43.2
GC content of SSC (%)	32.1	32.1	31.6	31.8	31.9	31.8	31.7	31.9	31.6
GenBank accession	OR187859	OR187860	NC_068050	NC_064998	NC_068051	NC_058571	MT422349	MT671999	NC_060687

### Analysis of codon preference

**Figure 3:**
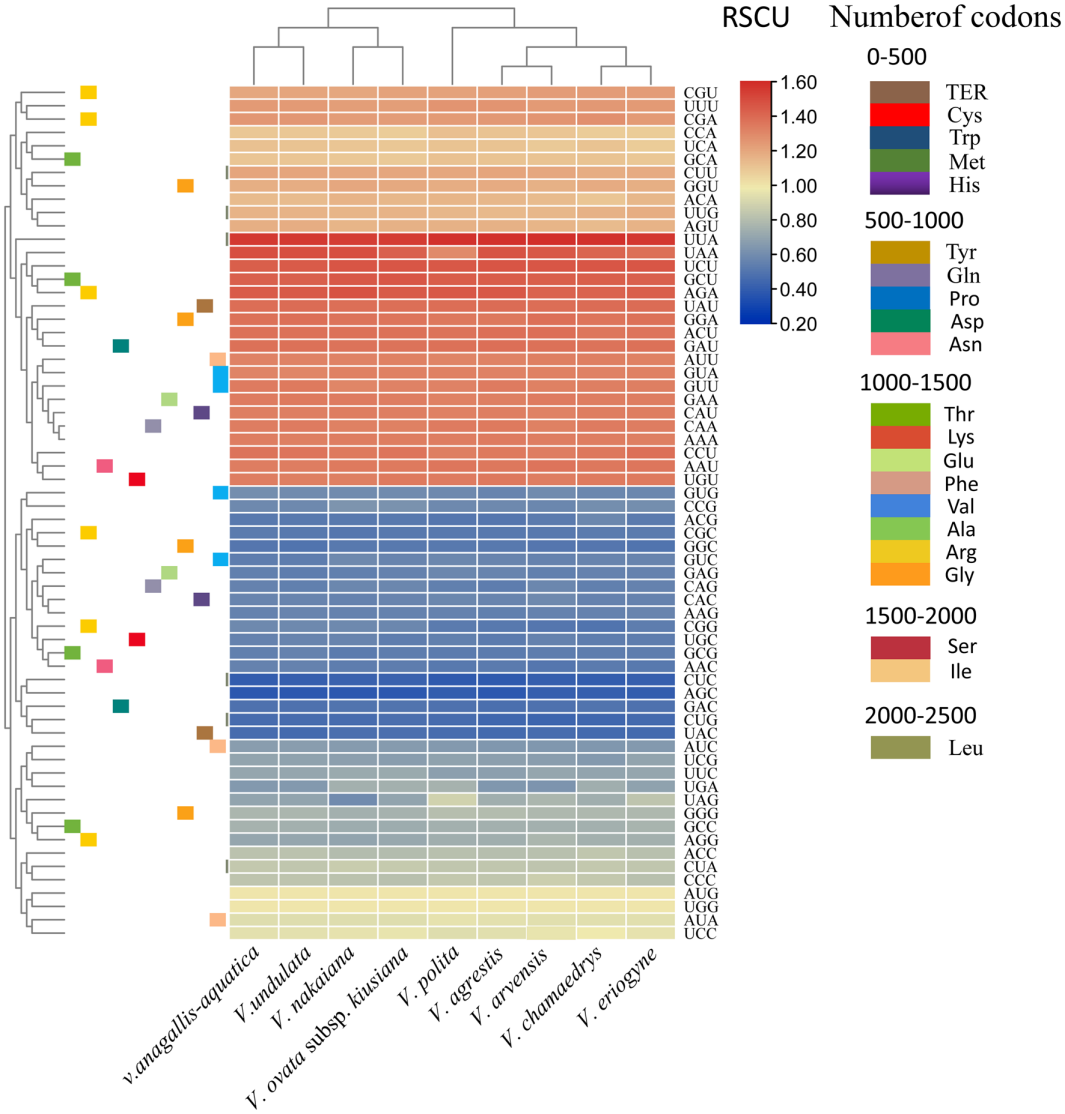
Heat map of RSCU values for nine *Veronica* species. Color key: the red values indicate higher RSCU values, and the blue values indicate lower RSCU values (For interpretation of the references to color in this Figure).

The analysis of codon bias revealed that *V. anagallis-aquatica* and *V. undulata* had 21,283 and 18,965 codons (including stop codons), respectively. Among all amino acid codons, the largest number of codons encoding leucine (Leu, L) was 2,277 and 2,281, followed by isoleucine (Ile) with 1,793 and 1,755, respectively. Cysteine (Cys, C) had the lowest number of codons with 226 and 227, respectively. In the cp genomes of the two species, there were 30 codons with RSCU > 1, accounting for approximately 50% of the total number of codons. Except for the codon UUG, all of them ended with A or U bases. The codon preference was also observed for 18 amino acid codons with RSCU ≥ 1.5. For instance, leucine (Leu) showed a preference for the codon UUA (RSCU = 1.95), arginine (Arg) for the codon AGA (RSCU = 1.73), and the termination codon for UAA (RSCU = 1.81). Out of the 32 codons with RSCU values less than 1, 29 of which were G/C-terminated codons. These results suggest that the cp genomes of the two species exhibited a preference for A/U-terminated codons over G/C-terminated codons. Both codons encoding methionine (Met, M) and tryptophan (Trp, W) had only one codon with a RSCU value of 1 and showed no preference for use (Table S2). In addition, the cp genome codon usage of medicinal *V. anagallis-aquatica* and *V. undulata* was similar. Compared with the other eight species, there was a significant difference in the use of cp genome codons in *V. polita* (Fig. 3).

### Repeated sequences and SSRs analysis

**Figure 4:**
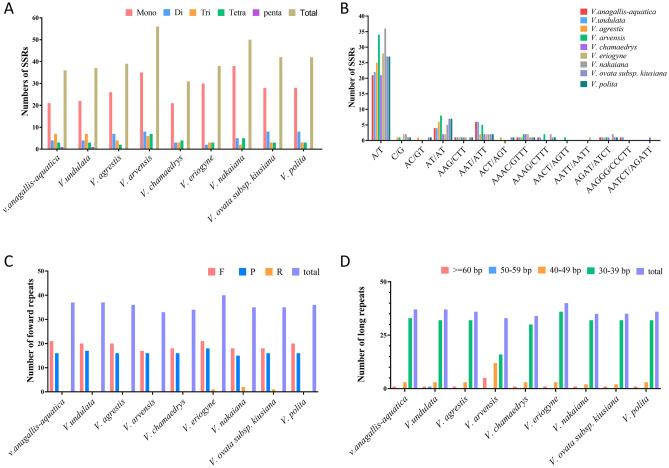
Analysis of SSR and long repeat sequences of 9 *Veronica* species. (**A**) The total number of SSR motifs. (**B**) The number of distinct types of SSRs. (**C**) Types of long repeats. (**D**) Length of the long repeats.

Long repeats are defined as repeated sequences with a length equal to or exceeding 30 bp. These facilitate cp genome rearrangement and increase a population’s genetic diversity. The distribution of the number of long repeats showed that 33–40 long repeats were identified in the cp genomes of 9 species. *V. eriogyne* had the largest number of long repeat sequences, while *V. arvensis* had the smallest number of long repeat sequences. The number of long repeat sequences of *V. anagallis-aquatica* and *V. undulata* was the same, both of which were 37 long repeats (Fig. 4C,D). The vast majority of long repeat sequence types are forward repeats and palindromic repeats. In 9 species, most of the long repeat sequences were 30–39 bp in length, and both had at least one long repetitive sequence more than 60 bp, respectively. No reverse repeat or complementary repeat sequence was identified in other species except for *V. eriogyne*, *V. nakaiana*, and *V. ovata* subsp. *kiusiana*(Table S3). SSR analysis showed that 36 SSR loci were detected in *V. anagallis-aquatica,* including 21 mononucleotides, 4 dinucleotides, 7 trinucleotides, 3 tetranucleotides, and 1 pentanucleotide, while 37 SSR loci were detected in *V. undulata,* including 22 mononucleotides, 4 dinucleotides, 7 trinucleotides, 3 tetranucleotides, and one pentanucleotide. *V. arvensis* had the highest total number of SSRs (56). No hexanucleotide repeats were found in the nine species, and single nucleotides often end in A/T (Fig. 4A,B and Table S4).

### Comparative analysis of the cp genomes of *Veronica*

**Figure 5:**
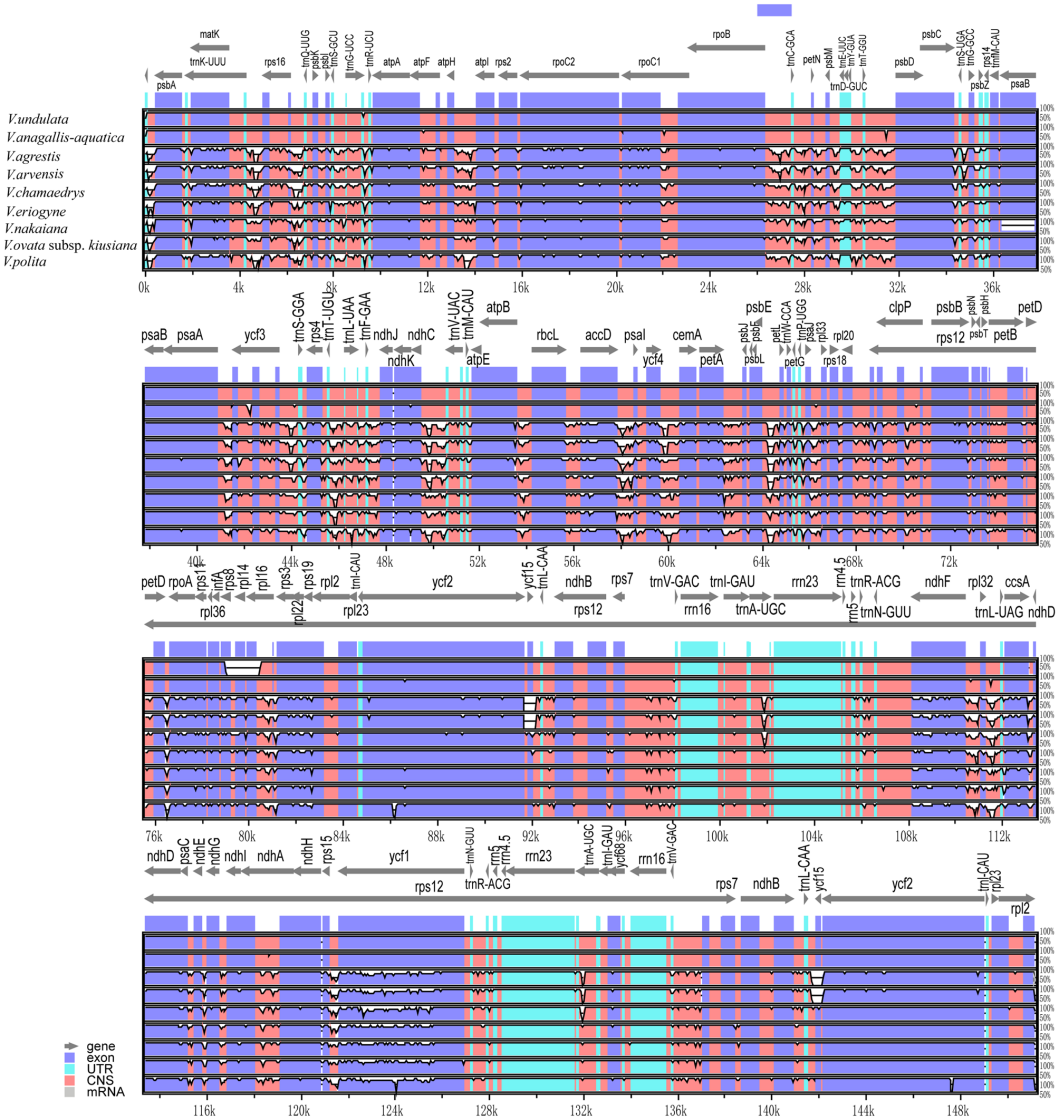
Comparison of cp genome sequences of 9 *Veronica* species. The x-axis represents aligned base sequences, and the y-axis represents percent pairwise identity within 50–100%. Purple represent exons, blue represent untranslated regions (UTR), pink represent non-coding sequences (CNS) and gray represent mRNA.

The mVISTA analysis showed that the level of variation of non-coding regions across 9 cp genome sequences was higher than that of conserved protein coding regions (Fig. 5). The variation in the LSC and SSC regions was significantly greater than that in the IR region, while the rRNA genes (*rrn4.5*, *rrn5*, *rrn16*, and *rrn23*) were highly conserved and had almost no variation. The sequence similarity between *V. anagallis-aquatica* and *V. undulata* was high, while the remaining seven species all differed significantly from the reference sequence. The highly differentiated regions in the 9 cpDNAs were mainly located in the intergenic spacers (IGS). For example, highly variable *trnT-GGU-psbD*, *ycf3*, and *rps8-rpl16* fragments could be used as potential molecular markers to identify *V. anagallis-aquatica* and *V. undulata*.

**Figure 6:**
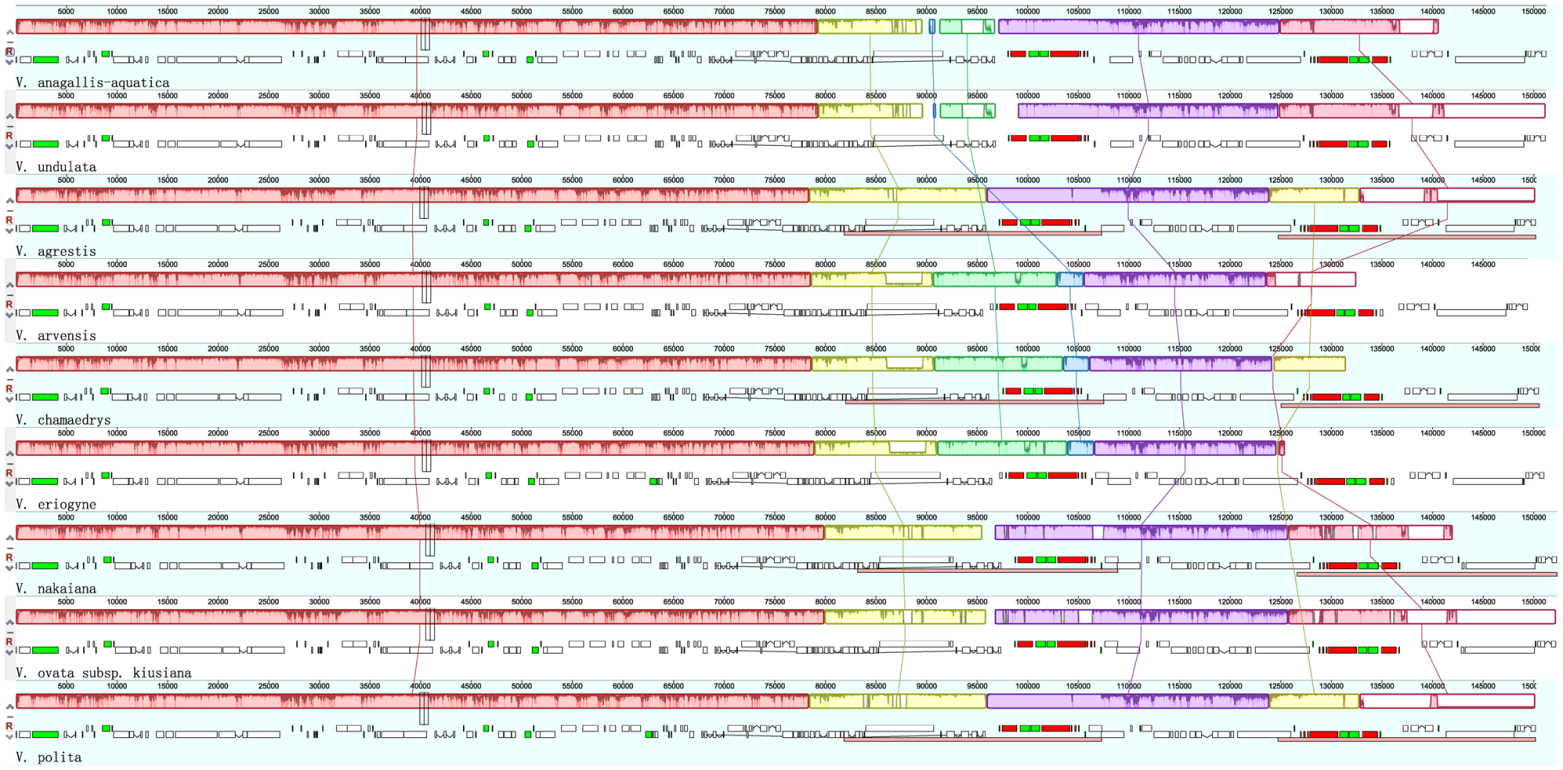
MAUVE alignment of 9 *Veronica* species chloroplast genomes using Geneious software. Within each of the alignments, local collinear blocks are represented by blocks of the same color and linked.

Homologous collinearity analysis was conducted on the chloroplast genome sequences of 9 *Veronica* species. The findings indicated that the purple region is a rearrangement region. The identical colored blocks linked by a common line symbolize analogous gene fragments from distinct species within the *Veronica* genus. All gene fragments are located above the axis, indicating that gene inversion has not occurred. The gene arrangement order of the chloroplast genomes of 9 species is consistent, with good collinearity, and there are no structural inversion phenomenon inside (Fig. 6). In terms of length, *V. undulata* is related to *V. agrestis* and *V. ovata* subsp*. kiusiana* is relatively close. In terms of location, *V. anagallis-aquatica*, *V. undulata, V arvensis*, and *V. eriogyne* has 5 common regions. It is worth noting that there were no significant deletions of gene fragments in the species of the *Veronica* genus, indicating that the cp genome sequences of *Veronica* plants are highly similar, and no inversion or gene rearrangement of large fragments were detected. Overall, these chloroplast genomes are essentially conserved and collinear.

### IR boundary analysis of cp genome in* Veronica*

**Figure 7:**
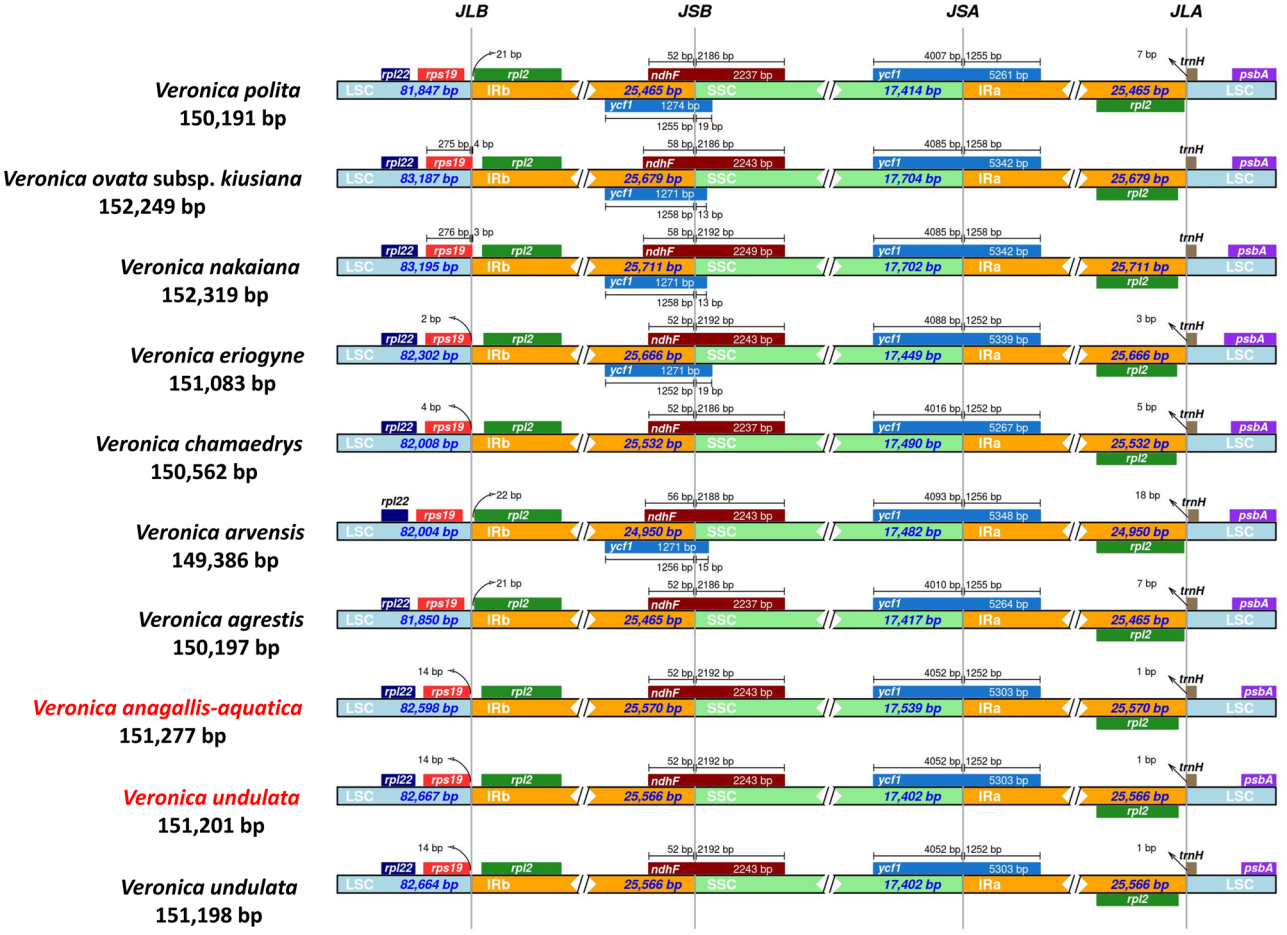
Comparison of the boundaries of LSC, SSC and IR regions in the cp genomes of 9 *Veronica* species. JLB connects LSC and IRb; jSB connects IRb and SSC; jSA connects SSC and IRa; jLA connects IRa and LSC.

The investigation into the shrinkage and expansion of the cp genome IR, LSC, and SSC boundary regions of nine species of *Veronica* indicates that the length of its cp genome is significantly different (149,386 bp-15,2319 bp). The *rpl2*, *rps19*, and *rpl22* genes were distributed in the boundary of LSC/IRb, and these nine species did not expand in this boundary. The *ycf1* and *ndhF* genes were distributed in the border IRb/SSC, and both were expanded. Notably, the *ycf1* gene was not detected at the IRb/SSC boundary in *V. anagallis-aquatica, V. undulata*, and *V. agrestis* (Fig. 7). The three genes of *rpl2*, *trnH*, and *psbA* were located at in the boundary of IRa/LSC, and no genes crossed this region. The SSC/IRa boundary of all 10 sequences was the *ycf1* gene, and expansion occurred. The length of the *ycf1* gene of *V. anagallis-aquatica* and *V. undulata* at this boundary was the same (5304 bp), while the length of the SSC region of the other seven species of *Veronica* was 4007–4093 bp. Similarly, the *rps19* gene expansion occurred in the LSC/IRb boundary for *V. ovata* subsp*. kiusiana* and *V. nakaiana*, while the other seven species of *Veronica* were located in the LSC region, with a distance of 14 bp away from the LSC/IRb boundary.

### Nucleotide polymorphism analysis of the cp genome

**Figure 8:**
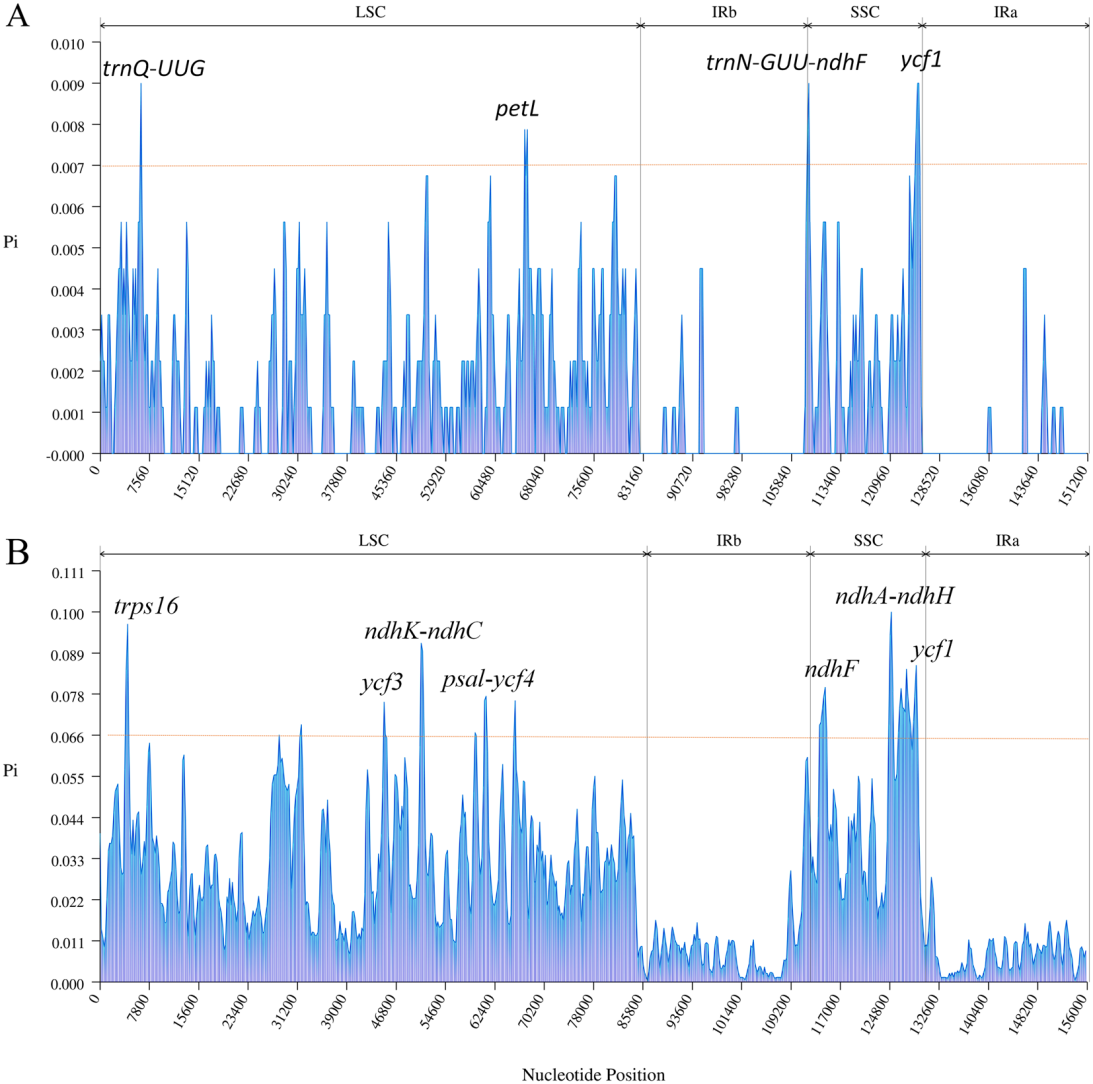
Sliding window analysis of cp genomes of *V. anagallis-aquatica* and *V. undulata* (**A**) and 9 *Veronica* species (**B**) (X axis: the position of the window 's midpoint; y axis : nucleotide polymorphism Pi value of each window).

The nucleotide polymorphism (Pi) results (Fig. 8A) showed that the Pi values for *V. anagallis-aquatica* and *V. undulata* were detected in the range of 1–151,418 bp (Table S5). The LSC and SSC regions showed higher variability in comparison to the IR region, which was consistent with the results of mVISTA analysis, indicating that the IR region behaved conservatively. In addition, the variation of these genomes were detected within a certain interval. Three highly variable regions were obtained, with Pi values greater than 0.007, including two intergenic regions (*trnQ-UUG* and *trnN-GUU-ndhF*) and two coding protein regions (*ycf1* and *petL*), which can be applied as hypervariable fragments to identify the above two species. The Pi of nine *Veronica* species (Fig. 8B) showed that 6 highly variable fragments with pi values greater than 0.066, including three intergenic regions (*ndhK-ndhC*, *psal-ycf4*, and *ndhA-ndhH*), and four genes (*rps16*, *ycf3*, *ndhF*, and *ycf1*). These regions can be potential molecular markers to identify nine different species of the genus *Veronica*.

**Figure 9:**
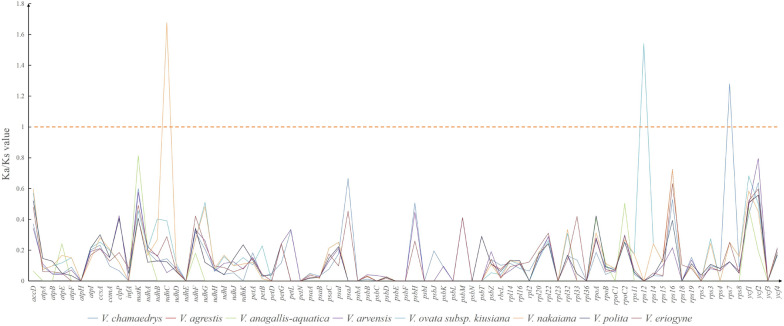
The Ka/Ks ratio of 79 protein-coding genes of 8 cp genomes for comparison with *V.undulata* (OR187860).

To assess the potential selection pressure on protein-coding genes in the genomes of the remaining eight species within the *Veronica* genus, non-synonymous (Ka) and synonymous (Ks) substitution rates, as well as Ka/Ks values, were computed based on the reference sequence of *V. undulata* (OR187860). The selection of Ka/Ks values results indicate that the average Ka/Ks value of protein genes under the eight genomes is 0.12309. Among all genes, except for *ndhC*, *rps12*, and *rps7* (Ka/Ks > 1) in some species, the Ka/Ks values of other genes are less than 1, indicating that the genes are in purification selection (Fig. 9) (Ka/Ks = 1, neutral selection, Ka/Ks < I, purification selection, and Ka/Ks > 1 positive selection). The Ka/Ks values of most genes are less than 0.6, indicating a clear purification selection pattern. Among them, up to 12 genes have Ka/Ks values of 0, indicating that these genes may be under strong purification selection pressure (Table S6).

### Phylogenetic and genetic distance analysis

**Figure 10:**
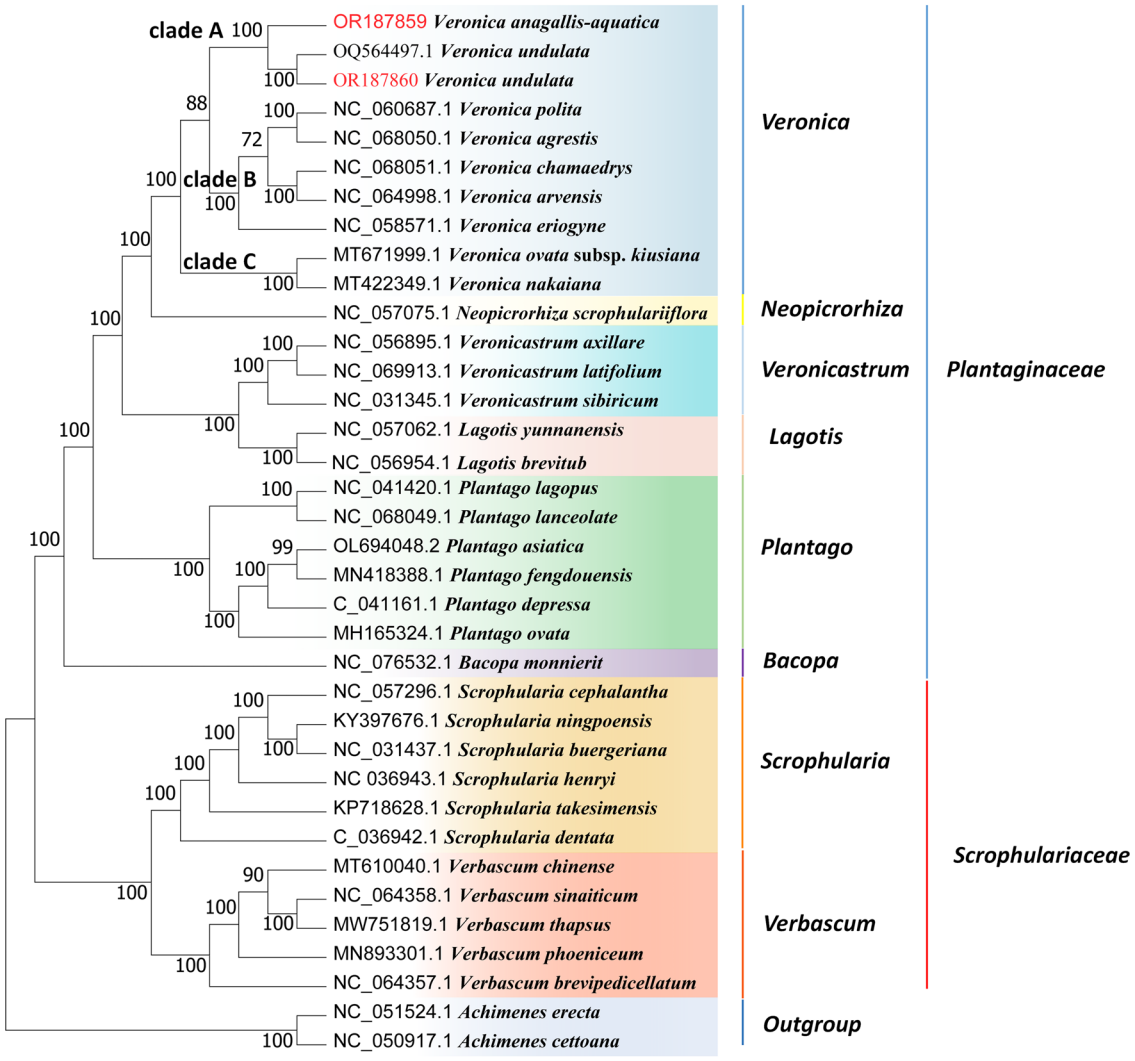
Maximum Likelihood (ML) phylogenetic tree based on complete cp genomes. *Achimenes cettoana* and *Achimenes erecta* were used as outgroups. Numbers at nodes are bootstrap support values.

**Figure 11:**
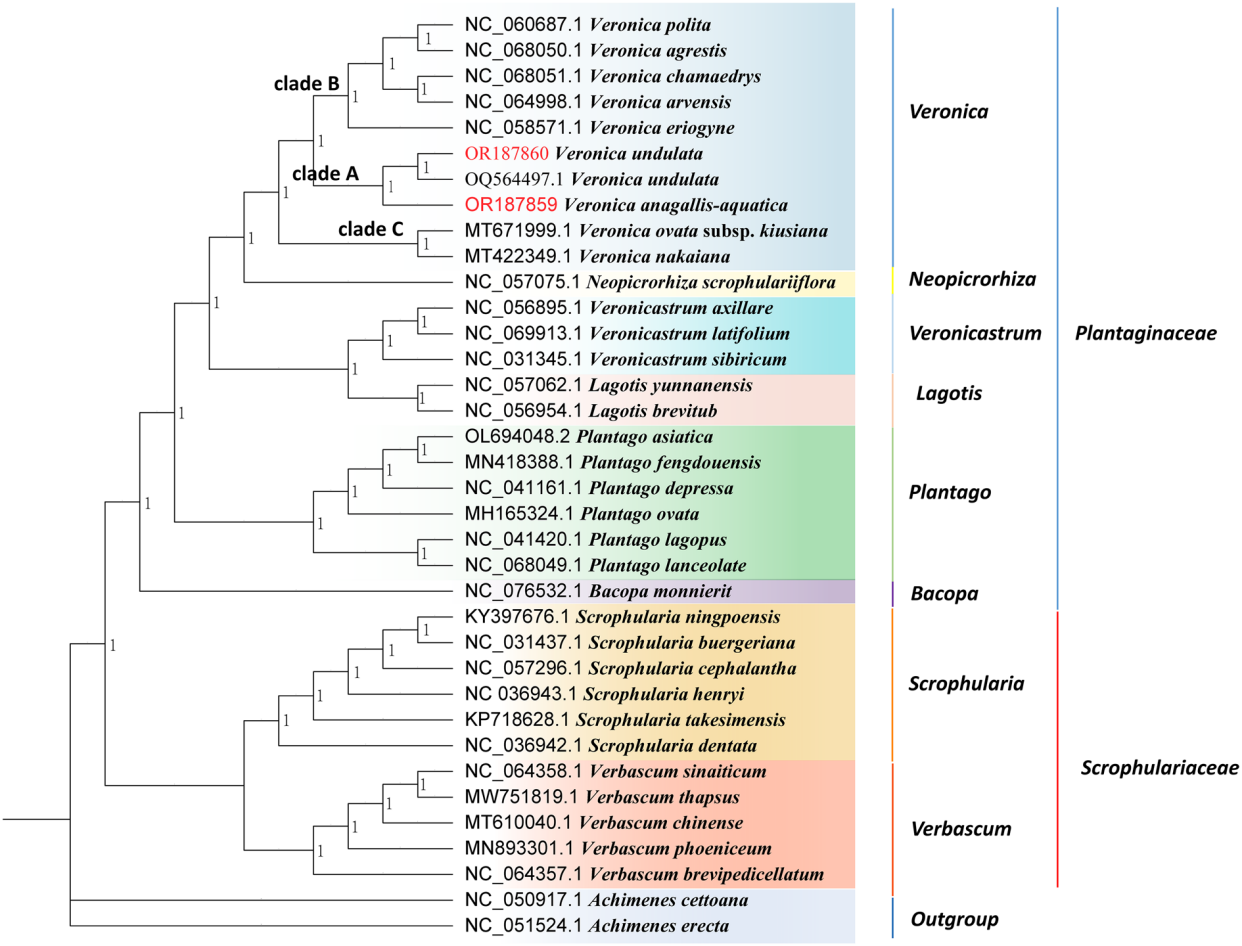
Bayesian Inference (BI) phylogenetic tree based on complete cp genomes. *Achimenes cettoana* and *Achimenes erecta* were used as outgroups. Numbers at nodes are bootstrap support values.

In the present study, we constructed ML tree (Fig. 10) and BI tree (Fig. 11) containing 36 complete cp genome sequences to determine the phylogenetic position of *V. anagallis-aquatica* and *V. undulata*. As depicted in the figure, most of the nodes exhibited high support values in the ML tree and BI tree, which is consistent with the support values of previous studies. The genus *Veronica* can be divided into three clades: (i) clade A, including *V. anagallis-aquatica* and *V. undulata*; (ii) clade B, including *V. polita* (NC_060687.1), *V. agrestis* (NC_068050.1), *V. chamaedrys* (NC_068051.1), *V. arvensis* (ON461915.1), and *V. eriogyne* (NC_058571.1); (ii) clade C, including *V. nakaiana* (NC_031153.1) and *V. ovata* subsp*. kiusiana* (MT671999.1). In addition, *Veronica* is clustered into one large clade with related species from the family Plantaginaceae, such as *Neopicrorhiza*, *Lagotis*, and *Veronicastrum.* This clustering highlights the close relationship between their genera. The relationship between the genera *Veronica* and Scrophulariaceae is far from *Scrophularia* and *Verbascum*, but close to the five genera of Plantaginaceae.

MEGA X v.10.2.6 software was utilized to assess the genetic distance of the complete cp genome of *Veronica* (Table 2). The results showed that the average genetic distance between the nine *Veronica* species ranged from 0.00040–0.042169, indicating that the genetic relationship between the *Veronica* samples was quite different. We found that the average genetic distance among *V. agrestis* and *V. polita* was the smallest, indicating that their genetic relationships were close. The average genetic distance difference between *V. anagallis-aquatica* and *V. undulata* is also small, indicating a close genetic relationship between them. Except for the four *Veronica* species mentioned above, there is a significant difference in the average genetic distance between other species.

**Table 2:** Analysis of the average genetic distance between species of K2-P model of cp genome of *Veronica* species.

	*V. agrestis*	*V. polita*	*V. chamaedrys*	*V. eriogyne*	*V. arvensis*	*V. nakaiana*	*V. ovata* subsp*. kiusiana*	*V. anagallis-aquatica*	*V. undulata*
*V. agrestis*									
*V. polita*	0.0000400								
*V. chamaedrys*	0.0280986	0.0280987							
*V. eriogyne*	0.0200895	0.0200763	0.0190025						
*V. arvensis*	0.0308235	0.0308101	0.0220150	0.0221219					
*V. nakaiana*	0.0336541	0.0336409	0.0333089	0.0246059	0.0360202				
*V. ovata* subsp. *kiusiana*	0.0334512	0.0334380	0.0329219	0.0243364	0.0357183	0.0032336			
*V. anagallis-aquatica*	0.0400129	0.0399996	0.0396377	0.0316905	0.0421686	0.0277542	0.0277107		
*V. undulata*	0.0397041	0.0396910	0.0390737	0.0311994	0.0416306	0.0273665	0.0271596	0.0021695	

### Divergence time analysis

**Figure 12:**
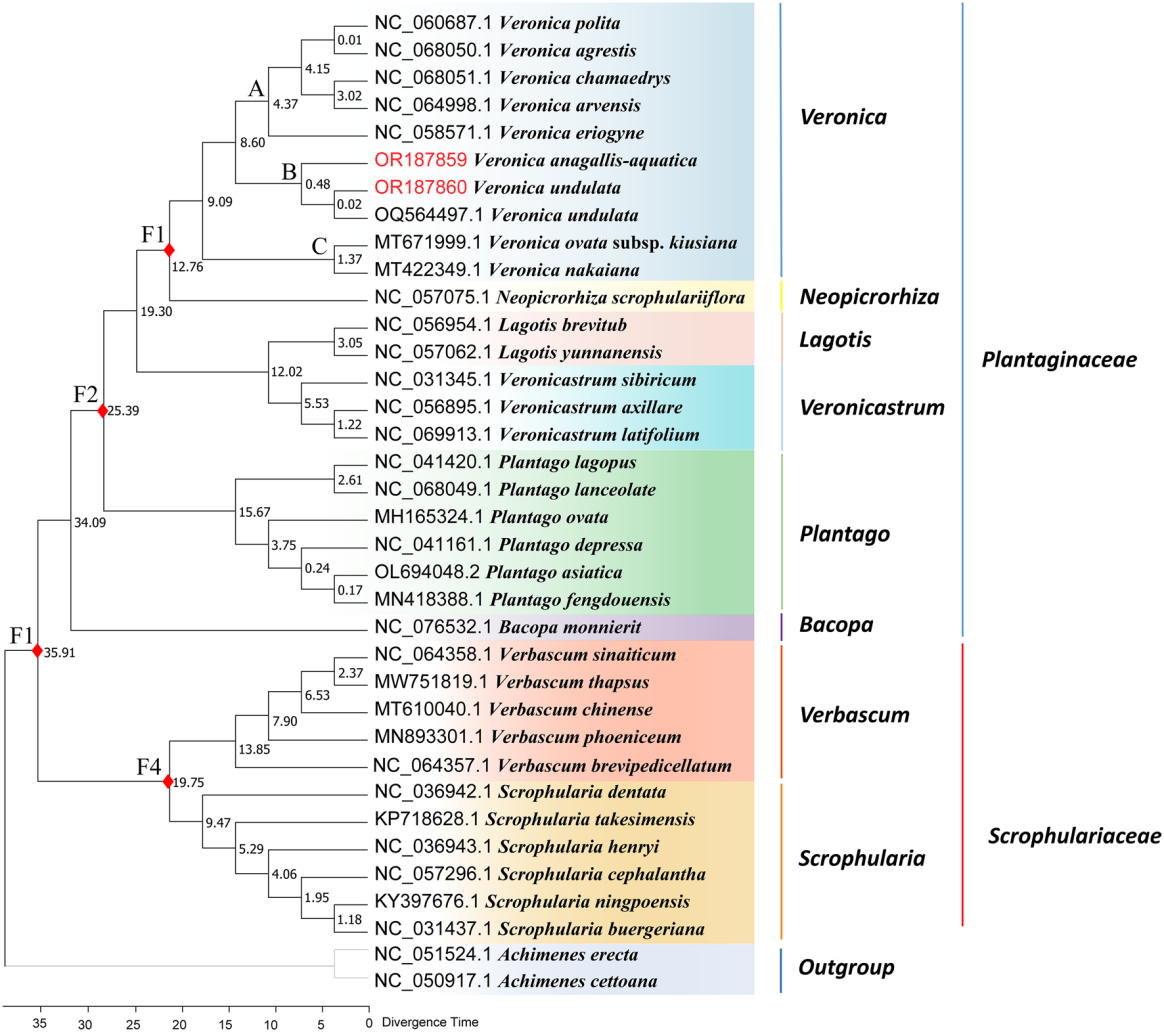
Divergence time estimation based on 36 cp genomes. The numbers next to the nodes represent the divergence time (Mya: millions years ago).

The species divergence times were estimated using 34 cp genomes and 2 outgroups. The results show that the ancestor of *Veronica* originated from about 12.76 million years ago (Mya) in the Oligocene, and began to differentiate at 9.9 Ma. There are two main lineages, clade A + clade B and clade C, which seem to have been dispersed since the Oligocene (clade A + clade B: 8.60 Ma; branch C: 1.37 Ma; (Fig. 12). *V. anagallis-aquatica* and *V. undulata* originated from 8.6 Ma and began to differentiate into two species at 0.48 Ma. In addition, the differentiation time of *Verbascum* and *Scrophularia* occurred at 19.75 Ma, and the differentiation time of Plantaginaceae and Scrophulariaceae occurred at 35.91 Ma.

## Discussion

### The cp genome structure and comparative analysis of the *Veronica* genus

The classification and identification of plants in the genus *Veronica* has tended to consider their morphological characteristics. However, geographical and ecological factors may continually change these characteristics in species of the genus *Veronica*^35,36^*.* Recent taxonomy studies have utilized cp genomes to assess the genetic relationship between related species^37–40^. The basic groups of nine species in this genus range in size variations from 149,386 to 152,319 bp, indicating that each of these species has significantly different cp gene sizes^13,41–43^. A comparative analysis of the cp genomes in *V. anagallis-aquatica* and *V. undulata* showed that they contain highly conserved structures and genes as well as the same protein-coding genes, tRNA, and rRNA. When using Illumina high-throughput sequencing and comparative genomics to analyze these cp genomes, the results demonstrated that some highly variable regions of these genomes can be correlational study-specific DNA barcodes for identifying species and analyzing phylogenetic relationships.

The GC content is an important indicator for determining phylogenetic interspecific relationships^44^. The total GC content, GC content in the IR, LSC, and SSC regions of *V. anagallis-aquatica* and *V. undulata* were consistent, while the other seven types of GC content had obvious differences. In addition, the *rps19* and *trnH* genes of both species were 14 and 1 bp away from the LSC/IR boundary, the *ndhF* and *ycf1* genes were both expanded, and the gene length was consistent, revealing the close relationship between these two species. Different IR boundaries exist between other species of *Veronica*, and the fluctuation of these boundaries is the main reason for interspecific differences^45^. Therefore, the GC content and boundary of the IR region to a high extent indicate that there is a close genetic relationship between *V. anagallis-aquatica* and *V. undulata,* but a distant phylogenetic relationship with other species of the genus *Veronica*.

Selection pressure refers to the pressure exerted by the outside world on a certain biological evolution process, which makes the species adapt to the natural environment^46^. Generally speaking, previous studies have shown that the Ka/Ks ratio is mostly less than one^47^. Because the synonymous nucleotide substitution rate is more common than the non-synonymous substitution rate. Genes with high Ka/Ks values can be used as candidate barcodes to distinguish species and will be applied in phylogenetic and systematic geographic analysis in the future^48^. This study shows that the Ka/Ks values of three genes (*ndhC*, *rps12*, and *rps7*) are all greater than 1, indicating positive selection.

### Repeat sequence and codon bias analysis

SSRs are repetitive sequences of 1–6 bp in length in the genome. These sequences are highly polymorphic, and those in non-coding regions are more variable than those in coding regions^49–51^. Currently, SSR analysis of cp genomes is widely applied in plant classification, biogeography, and population genetics^52,53^. We detected a total of 36 and 37 SSRs in the cp genomes of *V. anagallis-aquatica* and *V. undulata*, respectively. Most of the SSRs in both species are A/T mononucleotides. These results indicate that the cp genome SSRs consist mostly of adenine (A) or thymine (T) repeats, and few contain tandem guanine (G) or cytosine (C) repeats^54^. This conclusion resembles the results of studies on three Veroniceae species (*Veronica nakaiana*, *Veronica persica*, *Veronicastrum sibiricum*), most of which are A/T single nucleotide repeats^13^. Long repeat analysis of both species revealed only two repeat types (forward and palindromic), with the same number of repeats. It is noteworthy that *V. anagallis-aquatica* has one more forward repeat (F) than *V. undulata*, while *V. undulata* has one more palindrome repeat than *V. anagallis-aquatica*.

It is widely known that codon usage bias occurs in different organisms. Codon preferences are influenced by natural selection, base mutations, and genetic drift, are the result of long-term evolution of species in response to environmental selection, and affect the expression of mRNA and protein levels in the genome^55–59^. The most abundant amino acid in both species is leucine (Leu), with an average of 2,279, which is also common in other angiosperm species^60,61^ Meanwhile, based on the results of other angiosperm cp genomes, our study demonstrated that most codons ending in A/U have RSCU values higher than 1, which may be caused by a composition bias towards high A/T ratios^62,63^. Therefore, in the future, when designing the exogenous gene vector in *Boragina* spp. cp genetic engineering, the optimal codon ending with an A/U base is selected, which can appropriately improve the expression efficiency of exogenous genes. In addition, codon usage preferences were found as very similar in the cp genomes of these two species. These results may indicate that these two species experienced considerable environmental stress during their evolution.

### Phylogenetic analysis and Divergence time analysis

The cp genome is a valuable molecular tool in phylogenetic studies since it has a relatively conserved gene number and sequence. Furthermore, its taxonomic and evolutionary biological advantages, such as less susceptibility to recombination, have revitalized traditional fields such as taxonomy and evolutionary biology, and have extensively developed biological taxonomy^64^. For example, Zhang et al. (2018) applied the cp genome sequence of *Leonurus japonicus* to analyze its phylogenetic position, and their results showed that the genus *Leonurus* is more closely related to the genus *Stachys* in *Lamioideae*^65^*.* Cui et al. explored the phylogenetic position of *Zingiber officinale* in *Zingiberaceae* based on the published cp genome sequences of *Zingiberaceae* species. The results showed that *Zingiber* is a sister branch of *Kaempferia*^66^*.* Guo et al. first reported the cp genome sequence of *Schisandra chinensis*, and developed ML and BI phylogenetic trees to indicate that the genus *Schisandra* forms a sister group with the genus *Illicium*^67^*.*

*Veronica* is considered a relatively complex taxonomic group at both the morphological and molecular levels due to its large number of species, morphological similarities, and wide distribution areas. Previously, Wang et al. applied phylogeny to comparatively study perennial and annual plants of *Veronica*, and found that the ancestors of *Veronica* were likely perennial plants^68^. Albach et al. used ribosomal and plastid DNA sequences to reveal the phylogenetic relationships of the genus *Veronica* and its subgenera, finding that the Asian and European perennial species are monophyletic sister groups^69^. Ellmouni et al. phylogenetically analyzed the genus *Veronica* by using plastid and ribosomal DNA^70^. Xu et al.^71^ used the cp gene matK to divide 16 species of *Veronica* into two independent parts by establishing a phylogenetic tree, among which *Veronica hederifolia* was the most primitive. In this study, phylogenetic trees were constructed by ML, BI, MP (Fig. S1), and NJ (Fig. S2) methods using the cp genomes. The four phylogenetic trees showed the same evolutionary relationships, of which the genetic relationship between *Veronica* and *Neopicrorhiza* is the closest, followed by *Lagotis* and *Veronicastrum*, this is consistent with previous research results^41–43^. While that with related genus species under Scrophulariaceae is the farthest. Therefore, based on the phylogenetic relationship of cp genome, it is reasonable to classify *Veronica* into Plantaginaceae. In addition, two closely related species of *V. anagallis-aquatica* and *V. undulata* clustered in one clade with a bootstrap support value of 100%. This indicates that the cp genome can be used as a super barcode to identify *V. anagallis-aquatica* and *V. undulata*. Nevertheless, in this study, only one individuals were detected for each species, and further sample collection is needed for validation in the future to evaluate the discriminative power and effectuality of these loci in distinguishing diverse *Veronica* species.

Currently, the research foundation for estimating the divergence times of *Veronica* species is weak. This study found that the origin time of *Veronica* is about 9.09 Ma, and species diversity increased significantly during the Pliocene and Pleistocene. During the significant climate change of Cenozoic, the climate factors of the Tertiary and Quaternary periods had a great influence on the genetic structure and distribution of existing plants. The number of tropical plant species worldwide has decreased, while angiosperms have gradually increased, and plants from the Pleistocene period are similar to modern plants^72,73^. It is speculated that these climate changes may affect the diversity of *Veronica* plants and promote the radiation of the genus. However, as the genus with the highest number of species in the family Plantaginaceae, it is necessary to further evaluate the divergence times of the genus using broader molecular genetic data in the future.

## Conclusion

The two species of *V. anagallis-aquatica* and *V. undulata* have different functions and very similar morphologies. It is difficult to identify them by relying on traditional morphological classifications. In the present study, we compared and analyzed the cp genome characteristics of 9 species of the genus *Veronica*. The results indicate that the cp genome of the nine *Veronica* species exhibits a typical tetrad structure, with total lengths of 149,386 to 152,319 bp, and GC content of 37.9 to 38.1%. Comparative analysis showed that the cp genome sequences of *V. angalallis-aquatica* and *V. undulata* were less different, while the sequences of these two species were significantly different from those of the other seven genera of *Veronica*. Seven highly variable loci (*trnT-GGU-psbD*, *rps8-rpl16*, *trnQ-UUG*, *trnN-GUU-ndhF*, *petL*, *ycf3*, and *ycf1*) were identified as potential molecular markers to discriminate between these two species. The phylogenetic tree showed that *V. anagallis-aquatica* and *V. undulata* had a close genetic relationship and were sister groups. The molecular clock analysis results indicate that the divergence time of *Veronica* might occur at 9.09 Ma, and the divergence time of these two species occurs at 0.48 Ma. In conclusion, this study expanded the genomic resources of the genus *Veronica* as well as provided valuable information to support the phylogenetic analysis of this genus along with the identification of *V. anagallis-aquatica* and *V. undulata*.

## Materials and methods

### Materials, DNA extraction and sequencing

The plant materials used in this study were obtained from the field and were licensed for sample collection. Collection of plant materials is also in accordance with institutional, national or international guidelines. Fresh leaves of *V. angagallis-aquatica* (OR187859) were collected from Shuhe Town, Lijiang City, Yunnan Province, China (100°12′39″ E, 26°55′52″ N; elevation: 2403 m), and those of *V. undulata* (OR187860) were collected from Nanjian City, Yunnan Province, China (100°29′59.4″ E, 24°55′8.08″ N; elevation: 1927 m). The samples were identified by Professor Cong-long Xia (College of Pharmacy, Dali University) and Professor Wen-guang Yang (Kunming Institute of Botany, Chinese Academy of Sciences). The voucher specimens were preserved in the Herbarium of Dali University (Voucher Code of *V. angagallis-aquatica*: BSKM202201 and Voucher Code of *V. undulata*: SKM202202). Total DNA was extracted using the E.Z.N.A® Plant DNA kit (OMEGA). The chloroplast genome data of other *Veronica* species were downloaded from NCBI (https://www.ncbi.nlm.nih.gov/). Extracted DNA was checked for quality and integrity by 1% agarose gel electrophoresis, and also for concentration and content using TBS380 Picogreen (Invitrogen). Then, this DNA was fragmented to 300–500 bp using Covaris M220 sonication. The fragments were then purified using a TruSeq™ Nano DNA Sample Prep Kit for trimming, 3′-end adenylation, and ligation index adaptation. The sequencing library was created by conducting PCR amplification of appropriately-sized fragments, whose library was sequenced with paired-end (2 × 150 bp) using the Illumina NovaSeq 6000 platform (Shanghai Biozeron Biotech Co, Ltd).

### Genome assembly and annotation

Afterwards, the quality of paired-end Illumina^74^ reads was assessed with FastQC (http://www.bioinformatics.babraham.ac.uk/projects/fastqc/), and the low-quality reads were removed by using Fastp. GetOrganelle (v.1.6.4)^75^ was implemented to assemble the cp genome. Burrows Wheeler Alignment (BWA) tool was implemented to align high-quality reads to the cp genome sequence, and manual inspection was performed in the Integrative Genomics Viewer (IGV) tool to ensure that the assembly was correct. The complete cp genome sequence was annotated using cpGAVAS^76^ (http://47.96.249.172:16019/analyzer/annotate) and GeSeq^77^ (https://chlorobox.mpimp-golm.mpg.de/geseq.html), while the tRNA gene was identified and manually corrected by running tRNAscan-SE^78^. The cp genome map was drawn online using the Organellar Genome DRAW online tool^79^.

### Analysis of codon preference

Protein-coding genes with length < 300 bp and duplicates were removed, and CodonW (http://codonw.sourceforge.net) was applied to analyze codon usage^80^. Preference values of codons were obtained by calculating relative synonymous codon usage (RSCU). A codon RSCU value equal to 1.00 indicated that the codon lacked a preference, while an RSCU value greater than 1.00 indicated that the codon was used more frequently, and vice versa.

### Characterization of Repeat Sequences and SSRs

We used the online software REPuter (http://bibiserv.techfak.uni-bielefeld.de/ reputer/)^81^ to analyze the repeat sequences of cp genomes of the *Veronica* species. The parameter settings were as follows: The maximum calculated repeats were set to 5,000, the minimal repeat size was set to 30, the Hamming distance was set to 3, and other default parameters were set. MISA (https://webblast.ipk-gatersleben.de/misa/) detected markers of SSRs in the *Veronica* species, setting parameters as 10 for mononucleotide SSRs, 5 for dinucleotide SSRs, 4 for trinucleotide SSRs, as well as 3 each for tetranucleotide, pentanucleotide, and hexanucleotide SSRs^82^.

### Comparative analysis of cp genomes

By operating the online program mVISTA (https://genome.lbl.gov/vista/mvista/submit.shtml) to explore the differences between cp genomes within the genus *Veronica*, using the *V. undulata* (OQ564497.1) as the reference sequence, and setting the model of Shuffle-LAGAN to visualize and analyze the mVISTA structural variation map for nine *Veronica* species. The collinearity analysis of chloroplasts is a global alignment using the Mauve plug-in in Geneious v.9.0.2^83^ to perform collinearity analysis to detect gene rearrangements in the genome. The IRscope online tool (https://irscope.shinyapps.io/irapp/) was implemented to compare and analyze the structural variations of the IR region. The nucleotide diversity index Pi was calculated by sliding window analysis while using DNAsp software for *V. anagallis-aquatica*, *V. undulata*, and other *Veronica* species. The interspecific high variation sequences (hotspots) were screened based on the analysis results. The window length was set to 600 bp, and the step size was 200 bp^84^. The KaKs_Calculator v.2.0 software^85^ was used to calculate the substitution rate (Ka and Ks) of protein-coding genes.

### Phylogenetic and genetic distance analysis

Three species of *Veronicastrum*, 6 species of *Plantago*, one species of *Neopicrorhiza*, one species of *Bacopa*, 2 species of *Lagotis*, 9 species of *Veronica*, 6 species of *Scrophularia*, and 5 species of *Verbascum* were selected from the NCBI database (https://www.ncbi.nlm.nih.gov). *Achimenes cettoana* (NC_050917.1) and *Achimenes erecta* (NC_051524.1) were chosen as outgroups, and all sequences were subjected to multiple alignment of sequences by using MAFFT v.7.0^86^ Phylogenetic analysis was carried out using three tree-building methods, Neighbor-joining (NJ), Maximum likelihood (ML), Bayesian inference (BI), and Maximum Parsimony (MP). Using MEGA X v.10.2.6^87^ software to establish NJ and MP trees, the parameter settings are: bootstrap repeats 1000 times, NJ tree model selection Kimura2-parameter, MP tree search method selection Subtree-Pruning-Regrafting (SPR). At the same time, MEGA X software was used to calculate the average genetic distance between species by Kimura 2-parameter (K2-P) model. The ML phylogenetic tree of *Veronica* and other species was constructed using IQtree v.1.6.12 software^88^ The best nucleotide substitution model was screened using IQtree's built-in model finder. The bootstrap repetition value was 1000. After the program was completed, the software MEGA X v.10.2.6 was used to process the image of the ML tree. In addition, MEGA X v.10.2.6 software was used to determine the best matching alternative model, and the GTR + G model was selected. The Bayesian tree was constructed using MrBayes v.3.2.6^89^ software. The parameters were set as follows: The Markov Chain Monte Carlo (MCMC) algorithm was calculated for 1,000,000 generations, with sampling once every 1,000 generations, and the first 25% were discarded. The remaining trees were used to construct Bayesian trees with posterior probabilities.

### Divergence time analysis

To analyze the divergence time of the genus *Veronica* and its related genera, we constructed a molecular clock tree based on ML tree using MEGA X v.10.2.6 software. The online program TimeTree^90^ (http://www.timetree.org/) was used to obtain the divergence time of *Veronica anagallis-aquatica* and *Verbascum brevipedicellatum* (F1, 31.5–56.1 Mya), *Veronica anagallis-aquatica* and *Plantago ovata* (F, 20.7–43.2 Mya), *Veronica anagallis-aquatica* and *Neopicrorhiza scrophulariiflora* (F3, 1.9–13.9 Mya), and *Verbascum brevipedicellatum* and *Scrophularia cephalantha* (F4, 12.2–26.6 Mya) as calibration constraints.

### Supplementary Information

Supplementary Legends.

Supplementary Figure S1.

Supplementary Figure S2.

Supplementary Table S1.

Supplementary Table S2.

Supplementary Table S3.

Supplementary Table S4.

Supplementary Table S5.

Supplementary Table S6.

## Data Availability

The complete chloroplast genomes reported in this paper have been submitted to the NCBI database under the following accession numbers: OR187859 for *V. angagallis-aquatica* and OR187860 for *V. undulata*.
