# Supplementary material for: The chloroplast genomes of two medicinal species (Veronica anagallis-aquatica L. and Veronica undulata Wall.) and its comparative analysis with related Veronica species
Source: Sci Rep. 2024 Jun 17;14:13945. doi: 10.1038/s41598-024-64896-7 (PMC11183227; doi:10.1038/s41598-024-64896-7)
Supplement: Supplementary file 1 — Supplementary Legends. [file 41598_2024_64896_MOESM1_ESM.docx]

The chloroplast genomes of two medicinal species (*Veronica anagallis-aquatica* L*.* and *Veronica undulata* Wall.) and its comparative analysis with related *Veronica* species

Yonglin Hai^1,2^, Yan Qian^1,2^, Meihua Yang^1,2^, Yue Zhang^1,2^, Huimei Xu^1,2^, Yongcheng Yang^1,2,^**^*^** and Conglong Xia^1,2,^**^*^**

^1^ College of Pharmacy, Dali University, Dali 671000, China.

^2^ Key Laboratory of Yunnan Provincial Higher Education Institutions for Development of Yunnan Daodi Medicinal Materials Resources, Dali 671000, China.

^*^ Correspondence: Yongcheng Yang (15241821063@163.com) and Conglong Xia [(long7484@126.com)](mailto:(long7484@126.com))

**Supplementary Figure and Table**

Table S1 List of genes annotated in the cp genomes of *V. anagallis-aquatica* and *V. undulata*

Table S2 Codon usage preference statistics of 9 *Veronica* species.

Table S3 Statistics of long repeat sequences of 9 *Veronica* species.

Table S4 SSR statistics of 9 *Veronica* species.

Table S5 Pi.stat of 9 *Veronica* species and Pi.stat of 2 *Veronica* species.

Table S6 The Ka/Ks ratio data of 79 protein-coding genes in 8 cp genomes (compared with *V. undulata*).

Fig. S1 The MP phylogenetic tree is based on complete cp genomes. *Achimenes cettoana* and *Achimenes erecta* were used as outgroups. Numbers at nodes are bootstrap support values.

Fig. S2 The NJ phylogenetic tree is based on complete cp genomes. *Achimenes cettoana* and *Achimenes erecta* were used as outgroups. Numbers at nodes are bootstrap support values.
