# Supplementary figures and images for: The chloroplast genomes of two medicinal species (Veronica anagallis-aquatica L. and Veronica undulata Wall.) and its comparative analysis with related Veronica species
Source: Sci Rep. 2024 Jun 17;14:13945. doi: 10.1038/s41598-024-64896-7 (PMC11183227; doi:10.1038/s41598-024-64896-7)

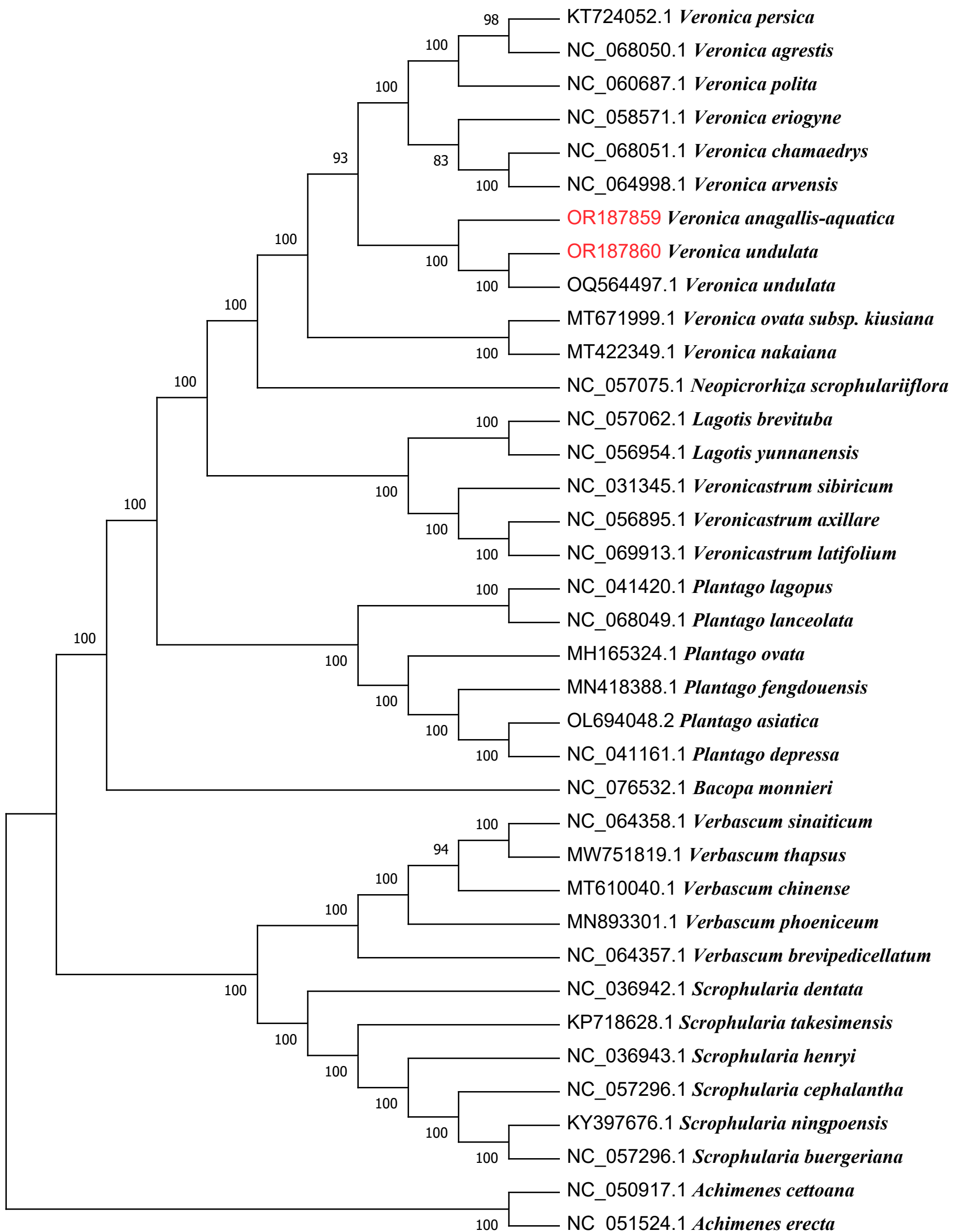

Supplement: Supplementary file 2 — Supplementary Figure S1. [file 41598_2024_64896_MOESM2_ESM.pdf]

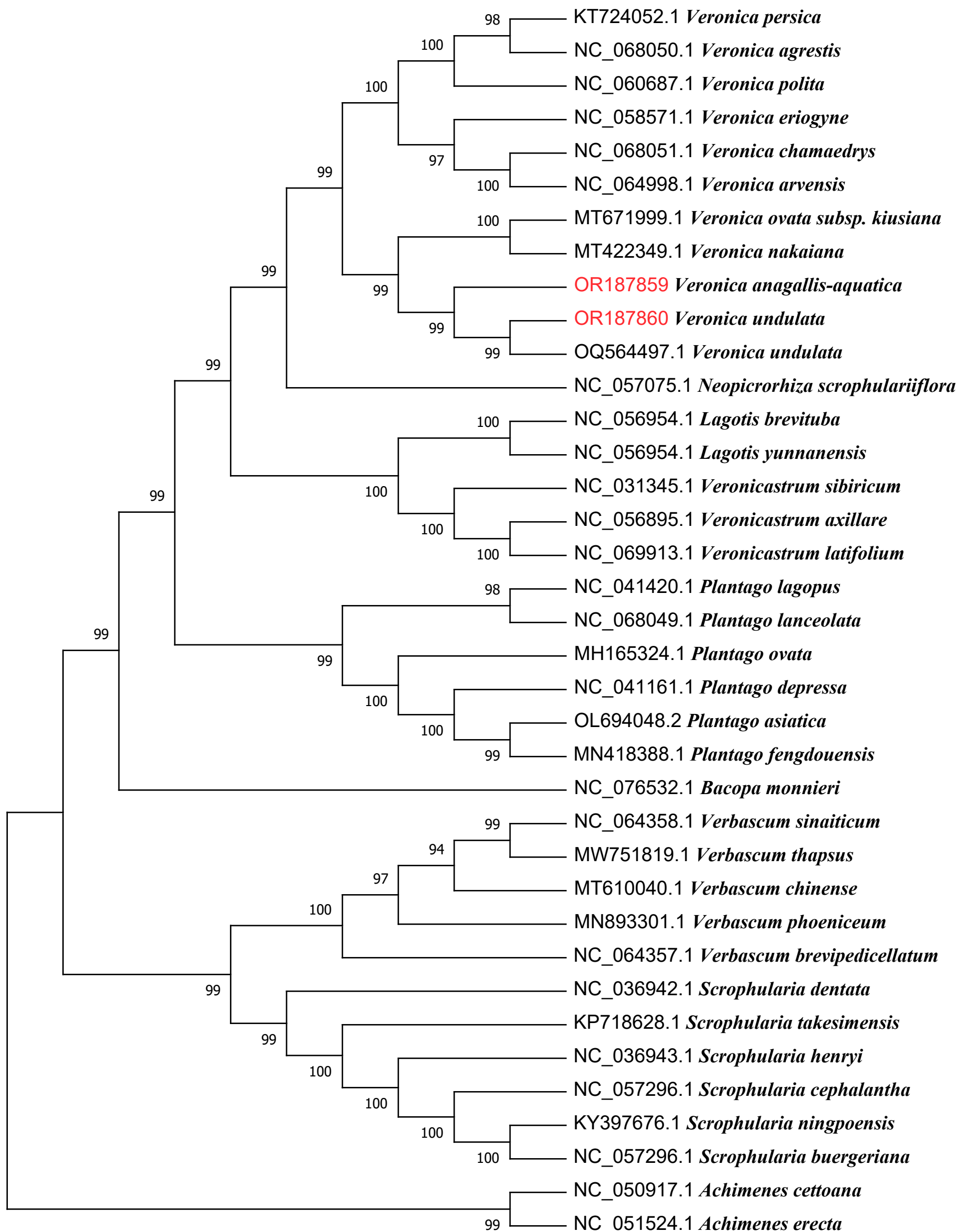

Supplement: Supplementary file 3 — Supplementary Figure S2. [file 41598_2024_64896_MOESM3_ESM.pdf]
